# A Novel Biallelic LCK Variant Resulting in Profound T-Cell Immune Deficiency and Review of the Literature

**DOI:** 10.1007/s10875-023-01602-8

**Published:** 2023-12-15

**Authors:** Anna-Lisa Lanz, Serife Erdem, Alper Ozcan, Gulay Ceylaner, Murat Cansever, Serdar Ceylaner, Raffaele Conca, Thomas Magg, Oreste Acuto, Sylvain Latour, Christoph Klein, Turkan Patiroglu, Ekrem Unal, Ahmet Eken, Fabian Hauck

**Affiliations:** 1 Division of Pediatric Immunology and Rheumatology, Department of Pediatrics, Dr. von Hauner Children’s Hospital, University Hospital, Ludwig-Maximilians-Universität München, Lindwurmstrasse 4, 80337 Munich, Germany; 2 Department of Medical Biology, Faculty of Medicine, Erciyes University, 38030 Kayseri, Turkey; 3 Molecular Biology and Genetics Department, Gevher Nesibe Genome and Stem Cell Institute, Betul-Ziya Eren Genome and Stem Cell Center (GENKOK), Erciyes University, Kayseri, Turkey; 4 Intergen, Ankara, Turkey; 5 Division of Pediatric Hematology & Oncology, Department of Pediatrics, Faculty of Medicine, Erciyes University, Kayseri, Turkey; 6 T Cell Signalling Laboratory, Sir William Dunn School of Pathology, Oxford University, Oxford, OX2 3RE UK; 7 Laboratory of Lymphocyte Activation and Susceptibility to EBV Infection, INSERM UMR1163, Paris, France; 8 Present Address: Hasan Kalyoncu University, Faculty of Health Sciences, Medical Point Hospital, Gaziantep, Türkiye; 9 Munich Centre for Rare Diseases (M-ZSELMU), University Hospital, Ludwig-Maximilians-Universität München, Munich, Germany

**Keywords:** LCK deficiency, inborn errors of immunity, profound T-cell immune deficiency, TCR signaling, CD4 and CD8 co-receptor expression

## Abstract

Lymphocyte-specific protein tyrosine kinase (LCK) is an SRC-family kinase critical for initiation and propagation of T-cell antigen receptor (TCR) signaling through phosphorylation of TCR-associated CD3 chains and recruited downstream molecules. Until now, only one case of profound T-cell immune deficiency with complete LCK deficiency [1] caused by a biallelic missense mutation (c.1022T>C, p.L341P) and three cases of incomplete LCK deficiency [2] caused by a biallelic splice site mutation (c.188-2A>G) have been described. Additionally, deregulated LCK expression has been associated with genetically undefined immune deficiencies and hematological malignancies. Here, we describe the second case of complete LCK deficiency in a 6-month-old girl born to consanguineous parents presenting with profound T-cell immune deficiency. Whole exome sequencing (WES) revealed a novel pathogenic biallelic missense mutation in *LCK* (c.1393T>C, p.C465R), which led to the absence of LCK protein expression and phosphorylation, and a consecutive decrease in proximal TCR signaling. Loss of conventional CD4^+^ and CD8^+^ αβT-cells and homeostatic T-cell expansion was accompanied by increased γδT-cell and Treg percentages. Surface CD4 and CD8 co-receptor expression was reduced in the patient T-cells, while the heterozygous mother had impaired CD4 and CD8 surface expression to a lesser extent. We conclude that complete LCK deficiency is characterized by profound T-cell immune deficiency, reduced CD4 and CD8 surface expression, and a characteristic TCR signaling disorder. CD4 and CD8 surface expression may be of value for early detection of mono- and/or biallelic LCK deficiency.

**Supplementary Information:**

The online version contains supplementary material available at 10.1007/s10875-023-01602-8.

## Introduction

Lymphocyte-specific protein tyrosine kinase (LCK) is critical for T-cell development and activation. LCK is recruited to the T-cell antigen receptor (TCR) after ligation of the TCR by peptide:MHC complexes on the surface of antigen-presenting cells [3, 4]. LCK-dependent phosphorylation of immunoreceptor tyrosine-based activation motifs (ITAMs) in the cytoplasmic tails of CD3δ, -ε and -ζ chains create docking sites for ζ -chain associated protein kinase 70 (ZAP70) [5, 6]. LCK next phosphorylates ITAM-bound ZAP70, which transduces the signal to the linker of activated T-cells (LAT). LAT, together with SRC homology 2 (SH2) domain-containing leukocyte protein of 76kDa (SLP76), forms a signal amplification and diversification hub, ultimately leading to T-cell activation [7, 8]. Besides the CD3-chains and ZAP70, LCK phosphorylates additional members of the TCR signaling network, such as protein kinase Cθ (PKCθ) and interleukin-2 inducible T cell kinase (ITK), and is involved in signaling downstream of other receptors, most prominently the co-stimulatory molecule CD28 [9], all together contributing to the central role of LCK in T-cell biology.

LCK differs from other SRC-family kinases (SFKs), such as FYN and SRC, in its N-terminal SH4 domain targeting LCK to the plasma membrane and the unique domain (UD) mediating (weak) interaction with the co-receptors CD4 and CD8 [10, 11]. SH3 and SH2 domains, providing docking sites for intra- and intermolecular interactions, are connected by a linker region to the kinase domain (KD) and a C-terminal unstructured tail. Inactive LCK, phosphorylated on Y505 by the C-terminal SRC Kinase (CSK) [12], adopts a closed conformation with an intramolecular interaction of pY505 with the SH2 domain [13]. De-phosphorylation of pY505 by CD45 [14–16] opens LCK into a primed state, which allows trans-autophosphorylation of Y394 resulting in full LCK kinase activity [17].

LCK is found both free and co-receptor bound with varying distribution between CD4^+^ and CD8^+^ T-cells and their differentiation stages [18], likely serving different purposes [19–21]. Despite CD4 and CD8 co-receptors being structurally dissimilar, the non-covalent interaction of both coreceptors with LCK is mediated by two conserved cysteines forming a Zn^2+^-clasp structure [10]. Importantly, while CD4 is present as a monomer, CD8 forms CD8αβ hetero- or CD8αα homodimers, in conventional or unconventional T-cells, respectively, where LCK is only bound to CD8α [22].

Besides LCK, a T-cell-specific isoform of FYN (FYN-T) and SRC are expressed in T-cells, albeit the latter only at low levels. Despite a high similarity between FYN-T and LCK, their functions are non-redundant, partly owing to different subcellular localization determined by their different SH4 domains [3]. Nonetheless, FYN-T can compensate for some LCK functions. While *lck*^-/-^ mice have an almost complete block in thymic positive selection, some peripheral mature T-cells develop [23]. In contrast, in *lck*^-/-^ *fyn*^-/-^ mice, no mature αβ T-cells are formed [24].

Owing to its critical role in T-cell biology, LCK loss of function (LOF) is expected to lead to a profound T-cell deficiency. Until now, only one case of complete LCK deficiency has been described [1]. A child suffering from recurrent, severe infections and immune dysregulation was found to have a biallelic missense mutation in *LCK* (c.1022T>C), resulting in an amino acid substitution in the kinase domain (p.L341P) with low protein expression and complete loss of LCK kinase activity. More recently, Li et al. described a homozygous splice site mutation in *LCK* (c.188-2A>G), predicted to affect the 3’ splice acceptor site of *LCK* exon 4, in a consanguineous family presenting with a partial CD4^+^ T-cell defect, and susceptibility to human papillomavirus (HPV) infections with atypical epidermodysplasia verruciformis (EV), as well as recurrent pneumonia [2]. Further, two reports of immune deficiency with reduced LCK protein expression and the presence of an aberrant isoform of *LCK* mRNA missing exon 7 have been published. However, pathogenic mutations in *LCK* were not identified in these reports, and signaling studies were not in line with defective LCK function [25, 26]. Lastly, beyond LCK, various inborn errors of immunity (IEIs) have been reported with deficiencies of proteins directly or indirectly activated by LCK, such as ZAP70 [27], LAT [28], SLP76 [29], ITK [30, 31] or components of the TCR-CD3 complex itself [32–35].

Here, we describe a novel complete biallelic *LCK* missense variant (c.1393T>C, p.C465R) in a patient with profound T-cell immune deficiency presenting with recurrent severe infections and characterize the molecular and cellular consequences of the variant for TCR signaling and T-cell function.

## Methods

### Human Samples

Blood samples were taken from the patient, relatives, and healthy volunteers who were treated at Mustafa Eraslan-Fevzi Mercan Children’s Hospital at Erciyes University and analyzed there and at the Dr. von Hauner Children’ Hospital at Ludwig-Maximilians-Universität München. Informed consent was obtained from both parents. This study was approved by Erciyes University local ethics committee (permit number: 2018/388) and conducted according to current ethical and legal guidelines and the Declaration of Helsinki.

### Sequencing

Whole exome sequencing (WES) was performed at Intergen NGS facility, Ankara, Turkey. DNA was isolated using a magnetic bead capture method (MagPurix, Zinexts). Exome enrichment was done using the Twist capture kit (TwistBiosciences). Sequencing was performed on a MGIseq DNBSEQ-G400 (MGI Tech Co.). Data was analyzed and interpreted following the ACMG criteria [36]. PCR amplification was performed via in-house designed primers. Amplicons were checked by 2% agarose gel electrophoresis. Confirmation sequencing was performed by the next-generation sequencing method by Miseq-Illumina equipment (Illumina, San Diego, CA, USA) according to manufacturers’ instructions. Data were evaluated with IGV 2.3 (Broad Institute) software. Sanger sequencing for genotype confirmation was done using the following primers: 5′-ACCTCTAGTGTGACCTTACCA-3′ (forward), 5′-GCAGAGTCCACGCAACTACA-3′ (reverse) following standard protocols.

### Lymphocyte Isolation and Cell Culture of T-Cell Blasts

Peripheral blood mononuclear cells (PBMCs) were isolated by density gradient centrifugation from peripheral blood samples using Ficoll-Paque Plus (Cytiva). PBMCs were frozen in human serum supplemented with 10% dimethyl sulfoxide (DMSO) in liquid nitrogen.

Primary T-cell blasts were generated from PBMCs of the patient and healthy controls (HCs). Frozen PBMCs stored in liquid nitrogen were quickly thawed and resuspended in prewarmed complete RMPI 1640 Glutamax (Invitrogen) supplemented with 10% FCS (Invitrogen) and Penicillin/Streptomycin 100U/ml (Invitrogen). PBMCs were stimulated with 5ng/ml phorbol-12 myristate-13-acetate (PMA) (Sigma), 1μM ionomycin (Sigma), and 200U/ml IL-2 (Novartis). After 2 days, cells were washed and cultured in complete RPMI with 100U/ml IL-2.

### Cloning and Plasmids

The 2nd generation lentiviral plasmid pCDH-CMV-insert-EF1a-LNGFR was provided by Dr. Thomas Magg, and the 3rd generation lentiviral plasmid pLJM1-EGFP[37] (Addgene #19319) was provided by Daniel Petersheim. The lentiviral helper plasmids psPAX2 (Addgene #10703) and pMD2.G (Addgene #12259) were provided by Dr. Oreste Acuto. Full-length LCK gene was amplified from the cDNA of healthy control PBMCs and subcloned into pCDH-CMV-insert-EF1a-LNGFR or pLJM1-EGFP. LCK-C465R was produced by site-directed mutagenesis using Q5 polymerase (New England Biolabs). A C-terminal HA-Tag was introduced into pCDH-LCK WT and C465R by PCR with a reverse primer specific for the C-terminus of LCK fused to a HA-Tag. All constructs were verified by Sanger sequencing.

**Table:** 

#	Primer	Sequence 5′-> 3′
1	pCDH-LCK FW	ATATCTAGAGCCACCATGGGCTGTGGCTGCAGCTCACACC
2	pCDH-LCK RV	ATAGCGGCCGCTCAAGGCTGAGGCTGGTACTGGCCC
3	pCDH-LCK-HA RV	ATAGCGGCCGCTCAAGCGTAGTCTGGGACGTCGTATGGGTA AGGCTGAGGCTGGTACTGGCCC
4	LCK C465R FW	GCATGGTGCGCCCTGACAACCGTCCAGAGGAGCTGTACCAA
5	LCK C465R RV	TTGGTACAGCTCCTCTGGACGGTTGTCAGGGCGCACCATGC
6	pLJM-LCK FW	ATAGCTAGCGCCACCATGGGCTGTGGCTGCAGCTCACACC
7	pLJM-LCK RV	ATAGAATTCTCAAGGCTGAGGCTGGTACTGGCCC

### Cells, Transfections, and Lentiviral Transductions

Cell lines were maintained at 37°C with 5% CO_2_ in a humidified incubator. Jurkat cells (clone E6.1, ATCC TIB-152), LCK-deficient Jurkat cells (J.CaM1.6, ATCC CRL-2063), and derived cell lines were maintained in RPMI 1640 Glutamax (Gibco) medium supplemented with 10% FCS (Invitrogen). The human embryonic kidney epithelial cells (HEK293) derivative Lenti-X 293T cells (Takara, Cat-No. 632180) were maintained in DMEM (Gibco) supplemented with 10% FCS and 4mM Glutamax (Gibco). Lentiviral particles were produced in Lenti-X 293T cells by co-transfection of the transfer plasmids pCDH or pLJM1 with the packaging plasmids psPAX2 and pMD2.G complexed with polyethylenimine (PEI, linear MW25K, Polyscienes, Cat-No. 23966-100). Forty-eight hours after transfection, viral supernatants were harvested, filtered, and used for the transduction of J.CaM1.6 cells in the presence of 5mg/ml polybrene. Twenty-four hours post-infection, cells were washed and re-suspended in RPMI 10% FBS. Forty-eight hours post-infection, puromycin selection was started on cells transduced with pLJM1 which contains a puromycin-resistance gene.

### Stimulation for Immunoblotting

Cells were rested for 15 min on ice in RPMI 0% FCS. For anti-CD3/CD28 stimulation, cells were incubated for 15 min on ice with 1μg/ml soluble anti-CD3 (clone OKT3, BioLegend) with or without 5μg/ml anti-CD28 (clone CD28.2, BioLegend) and washed once with RPMI 0% FCS. In primary cells, antibodies were cross-linked with 10μg/ml goat anti-mouse IgG (BD) for 15 min on ice. In Jurkat cells, crosslinking was not performed. Stimulation was initiated by shifting cells to 37°C for the indicated times (2, 5, 15 min). Alternatively, cells were stimulated for 5 min at 37°C with 10ng/ml PMA and 1μM ionomycin or left untreated. The specific LCK inhibitor A770041 (Axxon Medchem) served as a negative control in healthy control cells. Cells were incubated with 10μM A770041 at 37°C for 10 min. After stimulation, cells were centrifuged at 4°C, and pellets were lysed immediately.

### Cell Lysates and Immunoblotting

Stimulated or unstimulated cells were pelleted at 4 °C, and pellets were vigorously resuspended in ice-cold complete lysis buffer (50mM Tris-HCl (pH 7.6), 150mM NaCl, 10mM NaF, 1mM Na_3_VO_4_, 1% Triton X-100, proteinase inhibitor) for 15 min on ice. Lysates were cleared by centrifugation at 15,000×g, 4°C for 15 min. The leftover cleared lysates were boiled with Laemmli sample buffer (Sigma). For whole cell lysates of Jurkat E6.1, J.CaM1.6 and transduced cells for immunoblots for LCK expression, total protein was quantified by BCA Protein Assay Kit (Thermo Scientific). Lysates were separated by SDS-PAGE and transferred onto a nitrocellulose membrane. After a blocking step with 3% BSA TBS-T, immunoblotting was performed with the following antibodies: mouse anti-human FYN (clone E-3), mouse anti-human GAPDH (clone 6C5), mouse anti-human LCK (clone 3A5), mouse-IgGκ BP-HRP, rabbit-IgGκ BP-HRP, anti-mouse-IgG-HRP (all Santa Cruz Biotechnology) or rabbit anti-human pZAP70 pY319 (clone 65E4), rabbit anti-human polyclonal pSRC-family kinase (pSFK) pY416, rabbit anti-human pERK1/2 pT202/pY204 (clone 197G2), and rabbit anti-HA-tag (clone C29F4) (all Cell Signaling Technologies).

### Surface and Intracellular Antigen Flow Cytometry Staining

0.5 Mio cells were harvested and washed twice with 1ml FACS buffer (PBS + 2% FCS). Cells were fixed with 150μl pre-warmed fixation solution (BD Cytofix®, BD Biosciences) for 10 min at 37°C. For staining of intracellular antigens, fixed samples were washed twice in 150μl permeabilization buffer (BD Perm/Wash I, BD Biosciences), re-suspended in 150μl permeabilization buffer, and incubated at RT for 30 min. Permeabilized cells were stained in 50μl permeabilization buffer containing the respective antibody dilution. For fluorescent-conjugated primary antibody staining, samples were incubated for 2h at 4°C. When fluorescent-conjugated secondary antibodies were used, they were diluted in 50μl permeabilization buffer and added to cells for 30 min at RT in the dark. Cells were washed 3 times with 1ml permeabilization buffer after each staining and twice with 1ml FACS buffer before surface staining. For staining of surface-expressed LNGFR, mouse anti-LNGFR-PE was added in 50μl of FACS buffer and stained for 30 min at RT in the dark. Cells were washed 3 times in FACS buffer, and samples were acquired at a LSRFortessa flow cytometer (BD Biosciences). The following antibodies were used:

**Table:** 

Antigen	Species	Fluorochrome	Clone	Supplier
HA	Rabbit	AF488	C29F4	CST
LCK	Mouse	n.a.	3A5	Santa Cruz Biotechnology
Mouse IgG (H+L)	Goat F(ab’)_2_	AF647		CST
LNGFR (CD271)	Mouse	PE	ME20.4-1.H4	Milteny Biotec

### Calcium-Flux Assay

Cells were harvested and rested for 2 h at 37°C, 5% CO_2_ in RPMI 1640 Glutamax (Gibco) 10mM Hepes without FCS. Cells were labeled with 5μM Indo-1-AM (Invitrogen) in RPMI 10mM Hepes for 30 min in the dark at 37°C, 5% CO_2_. After 30 min, RPMI 10mM Hepes 5% FCS was added to remove excess Indo-1-AM. After 30 min, cells were washed twice in RPMI 5% FCS and stained with anti-LNGFR-PE for 30 min in RPMI 5% FCS at RT in the dark. Cells were washed twice in 5ml RPMI 5% FCS and resuspended in RPMI 5% FCS before being acquired at a LSRFortessa flow cytometer (BD Biosciences). For calcium-flux measurement, cells were acquired for 1 min to record the baseline before addition of mouse anti-CD3 (BioLegend, clone OKT3) and anti-CD28 (clone CD28.2, BioLegend) both 2μg/ml followed by crosslinking with 4μg/ml goat anti-mouse IgG after 2 min. After 9 or 10 min, 1μM ionomycin was added to achieve maximum calcium-flux. Data were analyzed using FlowJo V9 (TreeStar).

### Immunophenotyping of Peripheral Blood Mononuclear Cells

Patient’s and healthy control PBMCs were thawed and washed with PBS (Gibco). An antibody master mix was prepared in BD Brilliant stain buffer (BD Biosciences), and samples were stained for 15 min at RT. Sample acquisition was performed on a LSRFortessa flow cytometer (BD Biosciences), and data were analyzed using FlowJo V9 (TreeStar). The following antibodies were used:

**Table:** 

Antigen	Fluorochrome	Clone	Supplier
CD45	BV480	HI30	BD
CD3	BUV496	UCHT-1	BD
CD4	BUV395	RPAT4	BD
CD8	PE-Cy5	RPA-T8	BD
CD45RA	BUV737	HI100	BD
CD45RO	BB515	UCHL1	BD
CD25	PE	M-A251	BD
CD27	APC-R700	M-T271	BD
CD28	BB700	L293	BD
HLA-DR	BV711	G46-6	BD
CXCR3	PE-CF594	1C6	BD
CCR7	BV421	G043H7	Biolegend
CCR6	BV785	G034E3	Biolegend
CD127	APC	A019D5	Biolegend
CCR4	PE-Cy7	L291H4	Biolegend
CXCR5	BV605	J252D4	Biolegend
CD38	BV650	HB-7	Biolegend

Fixable viability stain 780 (BD Bioscience) was used for viability staining.

## Results

### Case Description of a Girl with Profound T-Cell Immune Deficiency

**Fig. 1:**
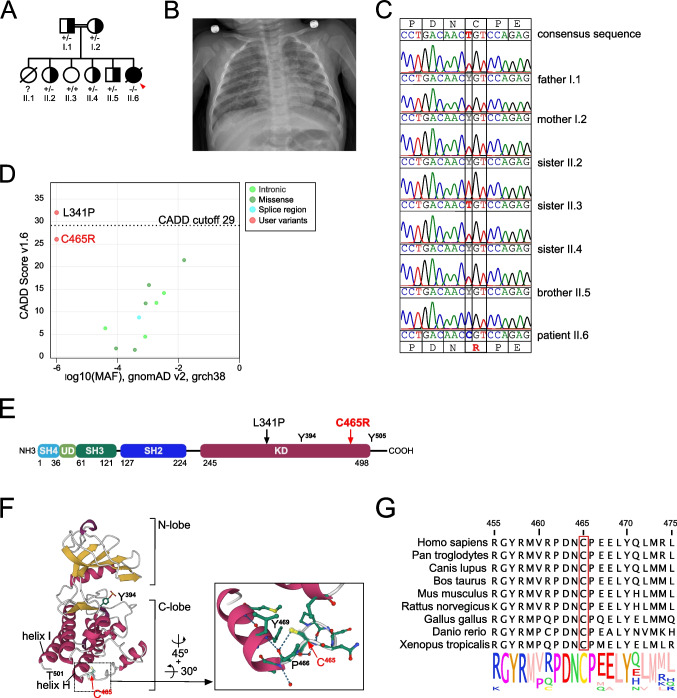
A novel biallelic missense variant in the LCK kinase domain. **A** Pedigree of the consanguineous family. The patient is indicated with an arrow. **B** Chest X-ray of the patient upon admission to Erciyes University Hospital in Kayseri. **C** Sanger sequencing of the index patient and indicated family members. Consensus amino acid and DNA sequence indicate on top, variant sequence below. **D** CADD vs. minor allele frequency (MAF)-plot of homozygous variants in *LCK* present in the gnomAD database and variants LCK C465R and LCK L341P [1]. Created with the PopViz webserver application [38]. The CADD cut-off was calculated with a 95% confidence interval. **E** Scheme of LCK protein: SH, Src homology region; UD, unique domain; KD, kinase domain. Numbers below indicate starting/ending aa residues of the individual domains. Arrows localize the variants L341P [1] and C465R. **F** (Right) Ribbon diagram of the X-ray structure of the kinase domain of human LCK in an open conformation (PDB ID: 3LCK) based on [17]. α-helices in purple, β-sheets in yellow, unstructured regions in grey. Residues Y394 and C465 are highlighted. (Left) Detailed view of the local structure surrounding C465. Carbon atoms depicted in green, nitrogen in blue, oxygen in red, and sulfur in yellow. Residues C465, P466, and Y469 are highlighted, and dashed lines indicate hydrogen bonds. Image created with Mol*Viewer [39] and RCSB PDB [40]. **G** Logo plot of LCK sequence conservation across the species indicated on the left. Created with Jalview [41]

The female patient was born at term to consanguineous parents of Syrian descent (Fig. 1A). There were 4 healthy siblings and one sister, who had died of respiratory failure due to a fulminant respiratory infection at the age of 7 months. The patient’s postnatal presentation, including body weight, body height, and head circumference, was unremarkable. Newborn screening had not been performed, because the patient did not have access to a newborn screening program. She developed normally until the age of 6 months, when she was admitted to a community hospital with fever and coughing, a diffuse maculo-papular rash, and oral and perianal candidiasis. After 1 month, she was transferred with progressive respiratory failure to Erciyes University, Kayseri, for further evaluation and treatment. The chest X-ray showed diffuse bilateral infiltrates (Fig. 1B). At the time of transferal, inflammation markers and leukocyte count were normal, but she had lymphocytopenia [lymphocytes 1,550/mm^3^ (N. 4,000–13,500)] and thrombocytosis [platelets 439,000/mm^3^ (N. 310,000 ± 68,000)]. Suspicion of an IEI was raised, and flow cytometric analysis of lymphocyte subsets revealed T-cell lymphocytopenia with an absolute reduction in helper and cytotoxic T-cells, while B- and NK-cells numbers were normal [CD3^+^ T-cells, 667/mm^3^ (N. 2,400–8,100); CD4^+^ T-cells, 289/mm^3^ (N. 1,400–5,200); CD8^+^ T-cells, 332/mm^3^ (N. 600–3,000)] (Table 1, row 1). High viral loads of cytomegaly virus (CMV) (6.2 × 10^6^ copies/ml), Epstein-Barr virus (EBV) (4.5 × 10^3^ copies/ml), and adenovirus (ADV) (16.4 × 10^6^ copies/ml) were detected in the blood and ganciclovir, and cidofovir treatment was initiated. In addition, because the patient had received BCG vaccination at the age of 2 months, treatment with rifampicin and isoniazid was started, but discontinued after 2 months because of hepatitis with elevated liver function tests (LFTs) (AST 1,166 U/l; ALT 632 U/l; GGT, 218 U/l; LDH 1,305 U/l). LFTs gradually decreased to baseline over the course of 4 weeks. While EBV and ADV became undetectable after 2 weeks of treatment, CMV viral load was still elevated after 40 days (3.6 × 10^6^ copies/ml). After exclusion of ganciclovir resistance, foscarnet was added, and after 2 weeks of consecutive combined treatment, a decline of CMV viral load could be observed (427 copies/ml). Overall, her clinical condition stabilized such that after 3 months of anti-infective treatment, she could be discharged with trimethoprim-sulfamethoxazole, fluconazole, and acyclovir prophylaxis and was scheduled for control at the pediatric hematology-oncology outpatient clinic. Allogeneic hematopoietic cell transplantation was offered but declined by the caregivers. Unfortunately, the family was lost to follow-up, and the patient died at the age of 12 months, 4 months after discharge, most probably due to respiratory failure following severe pneumonia.

**Table 1:** LCK variants described in the literature[1–4]

	Reference	LCK mutation	Age (at onset)	Sex	Protein levels	Clinical phenotype		CD3+ T-cells	CD4+ T-cells	CD8+ T-cells	CD19+ B-cells	IgM	IgG	IgA	IgE (reference range)	LCK signaling analysis	Scientific evaluation
							Outcome	mm3 or % (reference range)				g/l (reference range)					
1	Lanz et al. 2023	c.1393T>C, p.C465R	6 mos	F	Greatly reduced	Profound T-cell immune deficiency	Death due to respiratory failure	667 (2,400–8,100) 32.7 % (60-85)	289 (1,400–5,200) 14.2% (29-59)	332 (600–3,000) 16.3% (19–48)	828 (300–1,400) 40.6% (11-16)	0.19 (0.36–0.77)	4.86 (3.76–6.85)	0.11 (0.09-0.38)	0.37 U/ml (0-13)	Reduced Ca^2+^-flux, pZAP70 and pERK	Complete LCK deficiency
2	Li et al. 2016 [2]	Intronic c.188-2A>G (ΔExon3)	23 ys	F	Not shown	Atypical epidermo-dysplasia verruciformis with T-cell defect	Death due to invasive squamous cell carcinoma (SSC)	Not shown	Not shown, but reduced	Not shown	Not shown	Not shown			Not shown	Not shown	Suggestive of hypomorphic LCK deficiency with residual LCK WT expression
			18 ys	M			SSC, death, reason unknown										
			15 ys	F			Alive										
3	Hauck et al. 2012 [1]	c.1022T>C, p.L341P	18 mos	F	Greatly reduced	Profound T-cell immune deficiency	Death due to veno-occlusive disease d7 post-HSCT	1,254 (2,100–6,200)	154 (1,300–3,400)	1,012 (620–2,000)	506 (720–2,600)	3.06 (0.5–1.53)	8.7 (4.82–8.96)	1.21 (0.33–1.22)	<2 (<2 kUI/L)	Reduced Ca^2+^-flux and pTyr incl. pERK and pZAP70 after anti-CD3 stimulation	Complete LCK deficiency
4	Sawabe et al. 2001 [26]	Not identified. 2 LCK isoforms: WT & ΔExon7	22 ys	M	60% reduced	CVID	Alive, mild phenotype without treatment.	200 (n/a) 23% (58–78)	50 (n/a) 6% (33–52)	150 (n/a) 18% (119–34)	310 (n/a) 37% (3–13)	0.031 (n/a)	4.21 (n/a)	0.72 (n/a)	<29 U/ml (n/a)	Not shown	Not LCK-deficient. Expression of LCK ΔExon7 may be consequence of an unknown CVID.
5	Goldman et al. 1998 [25]	Not identified. 2 LCK isoforms: WT & ΔExon7	2 mos	M	Greatly reduced	SCID	HSCT	760 (2,000–7,035)	330 (1,520–4,830)	430 (640–2,730)	710 (800–3,600)	5 (33–155) 0.05 (3.3–15.5) ^a, b^	105 (252–708) 1.05 (2.52–7.08)^a, b^	6 (22–129) 0.06 (0.22–1.29)^a, b^	Not shown	Unperturbed Ca^2+^-flux and pTyr incl. pERK after anti-CD3 stimulation	Not LCK-deficient. Expression of LCK ΔExon7 may be consequence of an unknown SCID.

^a^Values at the age of 1 mo, before IVIG

^b^The original publication gives no units for Igs. According to the reference range given, the values refer to mg/dl

### A Novel *LCK* Variant Impairs LCK Protein Expression and Proximal TCR Signaling

The patient’s clinical presentation and immunological phenotype was suggesting a profound T-cell immune deficiency. Whole exome sequencing revealed a novel homozygous missense variant in exon 13 of *LCK* (HGNC:6524; c.1393T>C, p.C465R). Sanger sequencing confirmed a biallelic mutation in the patient, and both parents were found to be heterozygous supporting an autosomal-recessive inheritance pattern in this consanguineous family (Fig. 1C). Three of four healthy siblings (II.2, II.4, II.5) were heterozygous carriers, while one sister (II.3) was homozygous wildtype (Fig. 1C). The *LCK* variant was not present in the genome aggregation database (gnomAD) and predicted to be pathogenic by a combined annotation-dependent depletion (CADD) score [42] of 26.1 and a mutation significance cut-off (MSC) score [43] of 3.13. Only very few homozygous variants in LCK are found in gnomAD (Fig. 1D), none of them having similarly high CADD scores and low allele frequencies, and even heterozygous variants are rare (Fig. S1A). The gnomAD pLI score, which is a measurement of LOF-intolerance, is 0.99 for LCK, with a pLI ≥ 0.9 being LOF-intolerant [44], and a low observed/expected ratio (o/e) for pLOF variants (o/e = 0.067; 90% CI 0.027–0.21), suggestive of a haploinsufficient gene, is calculated [45]. For comparison, the pLI score for LOF variants in ZAP70 was 0.88 and the o/e ratio was 0.17 (90% CI 0.09–0.36)

The *LCK* variant resulted in an exchange of a small neutral cysteine to a large basic arginine (C465R) in a highly conserved region towards the end of the C-terminal lobe (C-lobe) of the KD [11] (Fig. 1E and F). C465 was not only conserved across species (Fig. 1G), but also in all members of the SRC kinases family (Fig. S1B), implying an important role for protein stability and/or function. In both open (PDB ID: 3LCK; Fig. 1F) and closed (PDB ID: 2pI0; Fig. S1C) forms of LCK, the sulfhydryl group of C465 forms a hydrogen bond with the neighboring residue P466, which in turn forms a hydrogen bond with Y469 in the α-helix H, and the introduction of an arginine was expected to disturb these interactions.

**Fig. 2:**
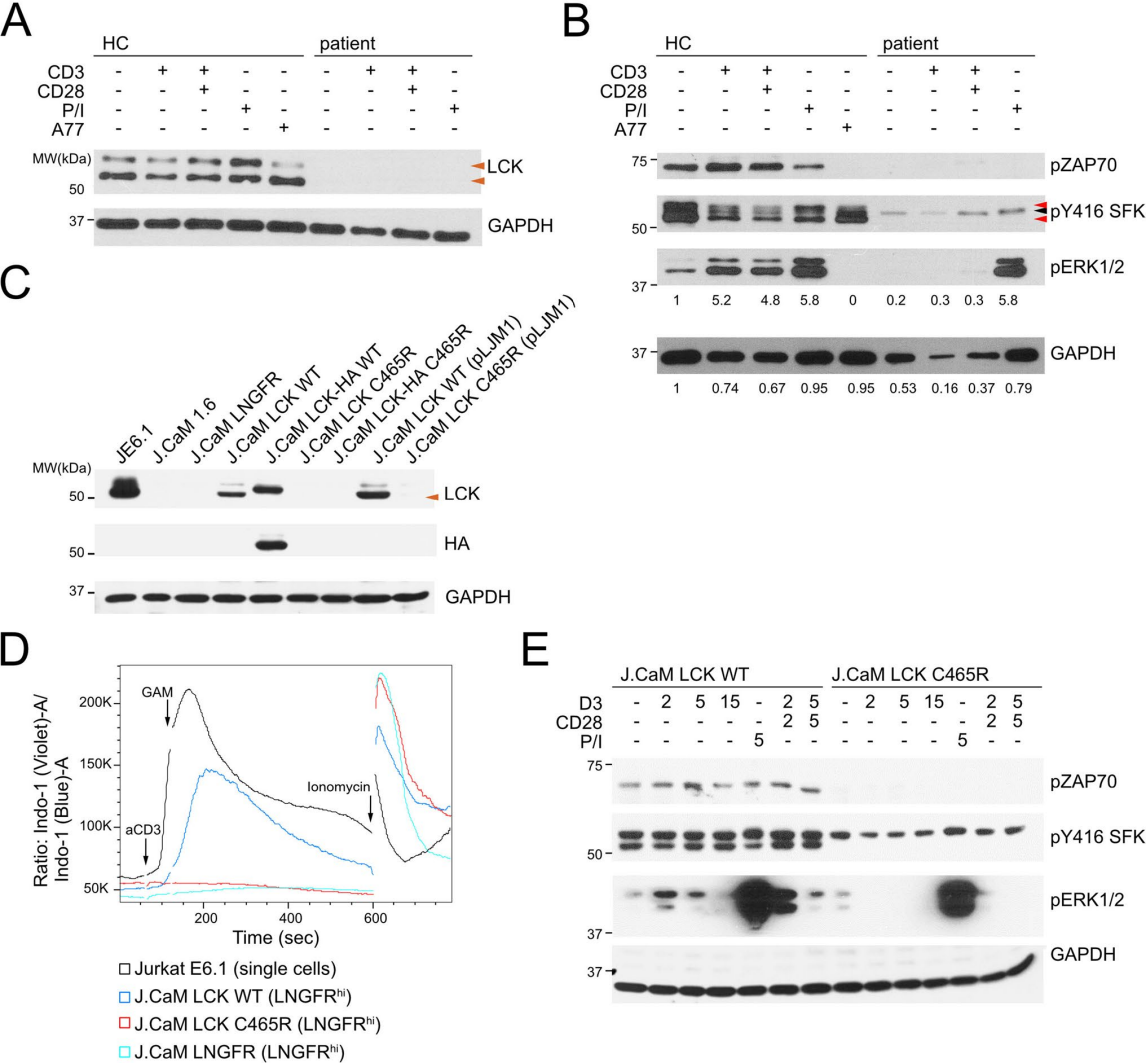
Reduced protein expression and TCR signaling due to LCK C465R. **A** LCK and GAPDH immunoblots of whole cell lysates of patient or healthy control (HC) T-cell blasts unstimulated or stimulated with anti-CD3 (2 min), anti-CD3/CD28 (2 min), PMA/ionomycin (P/I, 5 min) or treated with the selective LCK-inhibitor A770041 (A77, 10 min). Orange arrowheads indicate LCK bands. **B** pZAP70, pSFK (pY416), pERK1/2 or GAPDH immunoblots, conditions as in **A**. Red arrowheads indicate pLCK bands, black arrowheads pFYN-T. Numbers below pERK1/2 blot indicate fold change pERK1/2 band intensity (HC unstimulated =1), intensity normalized to GAPDH loading (numbers below GAPDH blot; GAPDH signal intensity normalized to HC unstimulated) **C** LCK, HA-tag and GAPDH immunoblots of whole cell lysates of Jurkat E6.1, J.CaM1.6, and stable transduced cells line expressing either LCK WT or LCK C465R with or without on C-terminal HA-tag or transduced with pCDH containing only the expression cassette for the extracellular domain of the low-affinity nerve growth factor receptor (LNGFR) (J.CaM EV). **D** Overlay of Ca^2+^-flux measurement in the indicated cell lines. Experiment representative of 3 independent experiments. **E** pZAP70, pSFK (pY416), pERK1/2 immunoblots in J.CaM1.6 cells transduced with pLJM1 containing LCK WT or LCK C465R and stimulated with either anti-CD3 (2, 5 or 15 min), anti-CD3/CD28 (2 or 5 min), PMA/ionomycin (5 min) or left untreated

Immunoblotting for LCK in T-cell blasts from the patient and a healthy control revealed absent LCK protein expression in the patient (Fig. 2A, orange arrowheads), suggesting that C465R influenced either protein expression or stability, or both. Active LCK is phosphorylated on Y394, corresponding to Y416 of SRC. As expected, immunoblotting with an anti-SFK pY416 antibody showed almost complete loss of active SRC-family kinases corresponding to the absent active LCK doublet in the patient T-cells (Fig. 2B, red arrows). A minor band detectable most likely represented residual FYN-T expression in patient T-cells (Fig. 2B, black arrow). Consistent with the absence of LCK protein expression, phosphorylation of ZAP70 was absent after stimulation with either anti-CD3 or anti-CD3/CD28 as compared to a healthy control (Fig. 2B). Very low residual levels of pERK1/2 (loading corrected pERK1/2 band intensity: HC unstimulated = 1, patient unstimulated = 0.2) were detectable in unstimulated patient cells that did not increase after stimulation with either anti-CD3 or anti-CD3/CD28 (Fig. 2B). Importantly, although LCK and ZAP70 phosphorylation was not rescued by stimulation with PMA/ionomycin (P/I), pERK1/2 levels were normal after P/I stimulation in patient T-cells as compared to the healthy control (loading corrected pERK1/2 band intensity: HC unstimulated = 1, HC P/I = 5.8, patient unstimulated = 0.2, patient P/I = 5.8) and inhibition with the LCK-specific inhibitor A770041 abrogated ZAP70 and ERK1/2 phosphorylation in T-cells of the healthy control (Fig. 2B). Collectively, these results suggested that the LCK C465R variant most severely affected LCK protein expression and LCK-dependent proximal TCR signaling in patient T-cells.

To verify the impact of the mutation on LCK expression and/or function in a model system, LCK wildtype (WT) or LCK C465R with or without a C-terminal HA-tag was expressed in LCK-deficient J.CaM1.6 Jurkat cells (J.CaM LCK WT or C465R) that were transduced with a lentiviral plasmid containing also the extracellular domain of the low-affinity nerve growth factor receptor (LNGFR, CD271) to identify transduced cells, or a puromycin-selectable plasmid (pLJM1). As in the patient cells, LCK C465R was poorly expressed in J.CaM1.6 Jurkat cells (Fig. 2C, orange arrowheads, and Figs. S2A and S2B). The defect in mounting a sufficient TCR signaling response was verified by Ca^2+^-flux measurements showing no response in J.CaM LCK C465R as opposed to J.CaM LCK WT (Fig. 2D and Figs. S2B and S2C). Immunoblotting for pY416 SFK, pZAP70, and pERK1/2 after stimulation with either anti-CD3 or anti-CD3/CD28 showed absent ZAP70 and LCK and ERK1/2 phosphorylation in J.CaM LCK C465R cells as compared to J.CaM LCK WT, while stimulation with P/I induced similar levels of pERK1/2 in J.CaM LCK C465R and J.CaM LCK WT (Fig. 2E and Fig. S2E), corroborating the defect seen in patient cells.

### Aberrant Immune Phenotype of Bi- and Monoallelic LCK-Deficient T-Cells

**Fig. 3:**
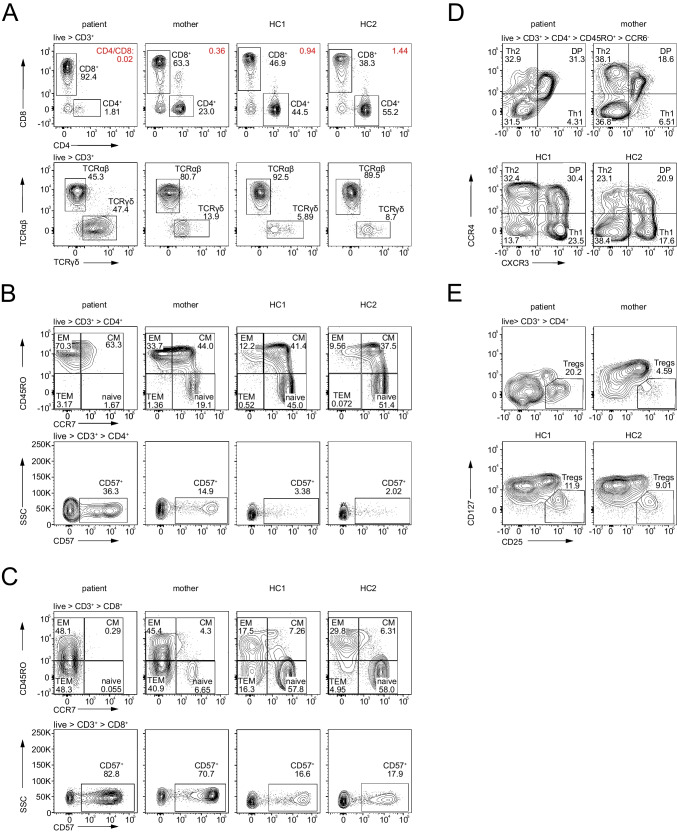
Immune phenotype of patient, mother, and two healthy controls (HC) by flow cytometry. Markers used for staining are indicated on the arrows next to the contour plots, gated populations above. For gating strategy, see Fig. S4. **A** (upper row) CD4 vs. CD8 expression on CD3^+^ lymphocytes, CD4/CD8 ratio in red; (lower row) αβ and γδT-cell frequency in CD3^+^ lymphocytes. **B** CD4^+^ T-cell differentiation (upper row) and CD57 expression (lower row). **C** CD8^+^ T-cell differentiation (upper row) and CD57 expression (lower row). **D** CD4^+^ CD45RO^+^ CCR6^-^ T helper cell phenotype. **F** Treg cells

To further characterize the impact of the LCK C465R variant, we performed immune phenotyping of cryopreserved PBMCs from the patient and her mother by flow cytometry. Two adult healthy controls were used alongside. We confirmed a severe loss of total CD3^+^ T-cells (patient 19.4% of CD45^+^ lymphocytes, mother 62%, HC1 58.6%, HC2 73.9%) and CD4^+^ T-cells (patient 1.81% of CD3^+^ lymphocytes, mother 23.0%, HC1 44.5%, HC2 55.2%) in the patient (Fig. 3A, upper row). CD8^+^ T-cells numbers were less affected, resulting in an inversed CD4/CD8 ratio of 0.02 (Fig. 3A, upper row). In addition, patient T-cells exhibited a reduction of CD4 and CD8 co-receptor surface expression, which has previously been described as pathognomonic for murine and human LCK deficiency [1, 23] (Fig. 3A and Figs. S3A and S3B). Unexpectedly, the heterozygous mother also displayed a reduction in CD4/CD8 ratio (0.36) and CD4 and CD8 co-receptor expression (Fig. 3A and Figs. S3A and S3B). Besides the decrease of CD4^+^ and CD8^+^ T-cells, the patient also showed a reduction of αβT-cell frequency with an increase of γδT-cells (Fig. 3A, lower row) expressing higher levels of TCRγδ on the surface as compared to healthy controls (Fig. S3B).

An almost complete loss of naïve CD4^+^ and CD8^+^ T-cells (1.67% and <1%, respectively) could be observed in the patient (Figs. 3B and C, upper rows). Most patient CD4^+^ T-cells were CD45RO^+^ CCR7^-^ effector memory T-cells (EM: 70.3%), but we also noted an increase in the terminally differentiated CD45RO^-^ CCR7^-^ population (TEM: 3.2 % vs HC1: 0.52% and HC2: 0.072%) that has been described to expand in chronic virus infections [46] (Fig. 3B). Patient CD8^+^ T-cells were evenly distributed between the effector memory and terminally differentiated effector cell compartment (EM: 48.2%; TEM: 48.3%) (Fig. 3C). Further, both CD8^+^ and CD4^+^ CD45RO^+^ memory T-cells in the patient were displaying an unusual CD38^hi^ HLA-DR^hi^ double positive state, most likely reflecting exhaustion [47–50] (Fig. S3C). As with co-receptor expression, we observed that the T-cells of the heterozygous mother displayed an intermediate phenotype between the T-cells of the patient and the two healthy controls with partial loss of naivety of both CD4^+^ and CD8^+^ T-cells (19% and 6.7%, respectively) and a concurrent increase in CD4^+^ EM T-cells, while CD8^+^ T-cells were equally distributed between EM and TEM (Figs. 3B and C). Additionally, CD57, a marker of chronic immune activation and senescence [51], was increased on both CD4^+^ and CD8^+^ T-cells in the patient (36.3% and 82.8%, respectively), but also in the mother (14.9% and 70.7%, respectively) (Fig. 3B and C, lower rows). Additionally, CD8^+^ T-cells had almost no CD28 surface expression, another sign of terminal differentiation and chronic immune activation and senescence [52, 53] (Fig. S3D).

Further, analysis of CD4^+^ helper T-cell subsets revealed a decrease of CCR6^-^ CXCR3^+^ CCR4^-^ Th1 cells in the patient and the mother (4.31% and 6.51%) (Fig. 3E). The percentage of CCR6^-^ CXCR3^-^ CCR4^+^ Th2 cells in the patient was like the healthy controls (Fig. 3E). Within the CCR6^+^ T-cell population, percentages of CCR4^+^ Th17 cells were comparable between patient, mother, and healthy controls (37.5%, 42%, 47.8% and 44.6%, respectively). However, because of a 50% reduction in CD4^+^ CCR6^+^ T-cells (Fig. S3E, upper row), the absolute number of Th17 cells was decreased in the patient. Furthermore, both patient and mother had very low percentages of CCR6^+^ CXCR3^+^ Th17.1 cells (5.0 and 8.43%) (Fig. S3E, lower row).

CD4^+^ CD25^hi^ CD127^lo^ Treg cell frequency was elevated in the patient (20.2%), although we noticed that CD25 expression within the Treg gate was relatively dimmer as compared with the healthy controls, and thus activated conventional T-cells may have been included in this gate [54] (Fig. 3F). Patient Tregs showed either an activated (CD45RO^+^ HLA-DR^+^) or memory (CD45RO^+^ HLA-DR^-^) phenotype (Fig. S3F).

Taken together, the LCK-deficient patient showed a severe absolute loss of total CD4^+^ and to a lesser extent of CD8^+^ T-cells with a relative increase in γδT-cells and Treg cells and a decrease of Th1 cells. Remaining CD4^+^ and CD8^+^ T-cells, including Treg cells, showed an activated, memory phenotype with an increase in CD57 and loss of CD28, indicative of immune senescence. Importantly, surface expression levels of the CD4 and CD8 co-receptors were reduced. Of note, the heterozygous mother showed an intermediate T-cell immune phenotype.

## Discussion

In the current study, we identified a novel biallelic missense *LCK* c.1393T>C, p.C465R variant in a patient from a consanguineous Syrian family with profound T-cell immune deficiency characterized by complete LCK protein expression deficiency and ensuing proximal TCR signaling- and CD4 and CD8-co-receptor-mediated functional and phenotypical defects. Both parents as well as three of four healthy siblings were monoallelic carriers of the *LCK* variant. Another child not amenable to analysis had passed away following a severe infection in the first year of life. Clinically, the patient presented in infancy with severe infections and passed away at the age of 12 months due to respiratory failure likely secondary to an infection, precluding more in-depth experimental analysis of primary T-cells.

We detected very low levels, if any, of the variant LCK by immunoblotting in patient cells and LCK-deficient J.CaM1.6 cells transduced with LCK C465R, suggesting reduced protein expression or stability, or both. However, we do not know the exact mechanism that led to reduced LCK protein expression, i.e., whether protein translation or folding was disturbed, or if the protein was subject to faster degradation. It is likely that the replacement of a small neutral cysteine with a larger basic arginine disturbed the local configuration with possible consequences beyond the local structure. Interestingly, the adjacent α-helices H and I form part of the interface of c-SRC with the kinase CSK that phosphorylates the inhibitory tyrosine Y527 (Y505 in LCK) [55]. For a better understanding of the structural changes induced by C465R and potential functional consequences, further analysis with molecular dynamic simulation of the variant compared to wildtype LCK could be employed.

To our understanding, this is the third LCK variant leading to a LOF phenotype to be described in the biomedical literature [1, 2]. The first reported LCK deficiency was due to a pathogenic *LCK* c.1022T>C missense variant, leading to the residual expression of a signaling incompetent LCK p.L341P with abrogated protein tyrosine phosphorylation and Ca^2+^-flux (Table 1, row 3) [1]. The patient presented early in life with profound T-cell immune deficiency characterized by severe infections, autoinflammation, autoimmunity, and ensuing failure to thrive. Li et al. reported 3 siblings of a consanguineous family presenting with recurrent pneumonia and severe viral skin disease leading to malignant transformation [2]. The patients had an intronic *LCK* c.188-2A>G splice site variant resulting in skipping of exon 3 and mRNA decay. Although the impact on LCK protein level, TCR signaling, and T-cell immune phenotype was not reported, genetic and clinical data alongside with CD4^+^ T-cell lymphocytopenia suggested a hypomorphic LCK deficiency (Table 1, row 2).

Two additional studies have shown defective LCK protein expression in the context of severe combined immune deficiency (SCID) and common variable immune deficiency (CVID), respectively [25, 26]. Goldman et al. reported a boy presenting with SCID with severe infections and profuse diarrhea (Table 1, row 5) [25]. He was lymphocytopenic with a greater reduction in CD4^+^ than CD8^+^ T-cells and reduced B-cells with hypogammaglobulinemia. CD8^+^ T-cells had absent CD28 surface expression and reduced LCK expression; however, TCR proximal protein tyrosine phosphorylation, Ca^2+^-flux and ERK-phosphorylation were unperturbed. cDNA analysis from PBMCs revealed the presence of *LCK* wildtype cDNA and an additional *LCK* transcript lacking exon 7 (*LCK ΔExon7*) that genetically and mechanistically remained unexplained. The adult CVID patient reported by Sawabe et al. had almost asymptomatic bihilar lymphadenopathy, reduced CD4^+^ T-cells, class-switched memory B-cells, and IgG (Table 1, row 4) [26]. Two *LCK* transcripts, corresponding to *LCK* wildtype and to *LCK ΔExon7*, were detected in the patient’s PBMCs, and reduced LCK protein expression was noted. Exon 7 encodes a part of the kinase domain including the ATP binding site necessary for kinase activity (NP_005347.3, aa 212-262). Indeed, Germani et al. [56] and we (Hauck and Latour, unpublished data) showed that LCK ΔExon7 protein or a variant with a mutation in the active site (K273E) lost its catalytic kinase activity. *LCK ΔExon7* is the sole transcript expressed in the J.CaM1.6 cell line that lacks LCK protein expression and has been generated under continuous mitogenic stimulation with PHA from Jurkat E6.1 cells [4, 57]. Low levels of the transcript coding for *LCK ΔExon7* are detectable in the parental Jurkat E6.1 cell line, thus the chronic PHA stimulation might have given a selective survival advantage to the LCK protein-deficient cells. Furthermore, *LCK ΔExon7* was shown to be expressed in PBMCs of healthy donors [58, 59]. Overall, we conclude that in both cases, the expression of *LCK ΔExon7* was probably not the cause, but the consequence of the underlying genetically unresolved SCID and CVID, respectively.

The immune phenotype of the LCK-deficient patient described here (LCK p.C465R) was very similar to that reported by Hauck et al. (LCK p.L341P) as well as to that of the *lck*^-/-^ mouse model [23, 60] with pronounced T-cell lymphocytopenia, inverted CD4/CD8 ratio, loss of T-cell naivety, exhausted memory phenotype in both CD4^+^ and CD8^+^ T-cells, and expansion of γδT-cells. However, we noted some differences between the two cases such as the percentage of Treg cells and the composition of immunoglobulin isotypes that may reflect either different functional consequences of the individual mutations or contact with different infectious agents and/or influences by additional genetic variants present in the patients. Immunologic workup of further LCK-deficient cases will help clarifying genotype-phenotype-correlations.

The immune phenotype of LCK deficiency reported here for the first time included T helper subsets showing a decrease of Th1, Th17, and Th17.1 cells. It is important to note, however, that due to the low frequency of CD4^+^ T-cells, the absolute numbers of events in the analysis of T helper subsets were low and analysis of further patients will give more clarity. The T helper subset phenotype is contrary to what has been reported from a mouse model with a post-thymic LCK gene deletion, which showed skewing towards Th1 responses in CD4^+^ T-cells [61], while peripheral T-cells in non-conditional *lck*^-/-^ mice have not been analyzed in such detail. This may be due to the differences in the environment, in particular the chronic viral infections that the reported patient has suffered from.

A prominent feature of human and murine biallelic LCK deficiency is reduced surface expression of the co-receptors CD4 and CD8 on T-cells [1, 23], and here we corroborated this finding. Additionally, we noted reduced co-receptor expression in an individual with monoallelic LCK deficiency, which previously has been described for CD4 in *lck*^+/-^ mice [23]. While CD4 internalization and degradation require phosphorylation of S408 by PKCθ and dissociation of LCK from CD4 to enable recognition of a dileucine motif by the clathrin adaptor AP2 [62–64], the mechanism for CD8 is less well understood and the shorter cytoplasmic tail of CD8 is devoid of both serin and dileucine motifs. This is in line with a recent study of a variant LCK unable to bind co-receptor, reporting differential regulation of CD4 and CD8 surface expression [21]. Thus, reduced LCK expression may increase CD4 endocytosis or by other mechanisms impede stabilization and reduce CD4 surface expression. Further experiments are needed to clarify the causes of reduced co-receptor expression in LCK deficiency, but we propose that it may be of value for early detection of mono- and/or biallelic LCK deficiency.

Besides reduced co-receptor expression, the mother with monoallelic LCK deficiency had further immune phenotypic alterations such as loss of CD4^+^ T-cells with an inverse CD4/CD8 ratio, CD4^+^ and CD8^+^ T-cell loss of naivety, and exhaustion. These changes could also be a sign of chronic viral infections, such as CMV [65]. Unfortunately, we were not able to acquire clinical information or blood samples for further analyses of the entire family. It is noteworthy that as opposed to ZAP70 or ITK deficiency, only one other case of complete LCK deficiency has been reported [1] and only two more are found in the ClinVar Database. The homozygosity in the case presented by Hauck et al. was due to a rare uniparental disomy; thus, only the mother was heterozygous for the LCK mutation [1]. Taken together, this raises the possibility that heterozygosity for a LOF variant in LCK may lead to clinical manifestations and purifying selection. Importantly, a recent study reported impaired proximal TCR signaling (pCD3 ζ, pZAP70, total-pY) in *lck*^+/-^ mice relative to WT mice [18]. Thus, it will be of interest to carefully screen individuals with immune dysregulation for monoallelic LCK deficiency in the future.

In summary, we report the second case of complete biallelic LCK deficiency causing profound T-cell immune deficiency with CD4^+^ and CD8^+^ T-cell lymphocytopenia and reduced CD4 and CD8 cell surface co-receptor expression. In individuals with suspicion for mono- or biallelic LCK deficiency co-receptor expression should be analyzed and could streamline immediate genetic workup.

### Supplementary Information

ESM 1 **A** CADD vs. minor allele frequency (MAF)-plot of heterozygous variants in *LCK* present in the gnomAD database and variants LCK C465R and LCK L341P[1]. Created with the PopViz webserver application[38]. The CADD cut-off was calculated with a 95% confidence interval. **B** Logo plot of sequence conservation across the human SRC-family kinases indicated on the left. Created with Jalview [41]. **C** (Right) Ribbon diagram of the X-ray structure of the kinase domain of human LCK in a closed conformation (PDB ID: 2PL0) based on [66] [17]. α-helices in purple, β-sheets in yellow, unstructured regions in grey. Residues Y394 and C465 are highlighted. (Left) Detailed view of the local structure surrounding C465. Carbon-atoms depicted in green, nitrogen in blue, oxygen in red and sulfur in yellow. Residues C465, P466 and Y469 are highlighted, and dashed lines indicate hydrogen bonds. Image created with Mol*Viewer [39] and RCSB PDB [40] (EPS 955 kb).

ESM 2 Supplementary Figure S2 **A** Histogram overlay of flow cytometry staining of Jurkat E6.1, J.CaM1.6 and stable transduced celllines with pCDH expressing either LCK WT or LCK C465R with or without an C-terminal HA-tag or transduced with pCDH empty vector containing only the expression cassette for the extracellular domain (ED) of the low-affinity nerve growth factor receptor (LNGFR) stained with anti-LNGFR-PE, anti-LCK + secondary anti-mouse AF647 and anti-HA-AF488. **B** Histogram overlay of flow cytometry staining of Jurkat E6.1, J.CaM1.6 and stable transduced celllines with pLJM1 expressing either LCK WT or LCK C465R transduced with empty vector pLJM1-EGFP anti-LCK + secondary anti-mouse AF647 and anti-HA-AF488. **C** Gating strategy used for Ca^2+^-flux measurements. J.CaM1.6 cells transduced with the lentiviral plasmid that co-expresses the extracellular domain (ED) of LNGFR were gated on LNGFR^hi^ for Ca^2+^-flux measurements, while for untransduced Jurkat E6.1 that do not express LNGFR-ED single cells were acquired. **D** Ca^2+^-flux measurements in J.CaM1.6 expressing LCK WT (light blue), LCK-HA (dark blue), LCK C465R (light red), LCK-HA C465R (dark red), Jurkat E6.1 (black) and J.CaM1.6 transduced with empty vector (turquoise) stimulated with anti-CD3 (left) or anti-CD3/ anti-CD28 (right). **E** pZAP70, pSFK (pY416), pERK1/2 immunoblots in Jurkat E6.1 cells or J.CaM1.6 transduced with empty vector pLJM1-EGFP and stimulated with either anti-CD3 (2, 5 or 15 min), anti-CD3/CD28 (2 or 5 min), PMA/ionomycin (5 min) or left untreated. (PNG 704 kb)

High resolution image (TIFF 3822 kb)

ESM 3 Supplementary Figure S3 Immune phenotype of patient, mother and two healthy controls (HC) by flow cytometry. Markers used for staining as indicated on the arrows next to the contour plots, gated populations above. **A** Contour plot overlay of CD4 vs. CD8 staining of patient, mother, and healthy control (HC)1 (all in red) with HC2 (blue) **B** Histogram overlays of the patient, mother and 2 healthy controls for surface expression of CD4, CD8, TCRαβ and TCRγδ. **C** CD38 vs. HLA-DR staining on CD45RO^+^ memory CD4^+^ (upper row) and CD8^+^ (lower row) T-cells of the patient, mother and 2 healthy controls. **D** CD28 vs. CD27 staining on CD8^+^ T-cells, color-coded by CD45RA expression **E** (upper row) CCR6 expression on CD4^+^ CD45RO^+^ T-cells and (lower row) CD4^+^ CD45RO^+^ CCR6^+^ T helper cell phenotype **F** Treg phenotype characterized by CD45RO and HLA-DR expression. (EPS 2842 kb)

ESM 4 Supplementary Figure S4 Gating strategy used for flowcytometric immune phenotyping. (EPS 596 kb)

## Data Availability

The datasets generated during and/or analyzed during the current study are available from the corresponding author on reasonable request.
