# Correction to: A Novel Biallelic LCK Variant Resulting in Profound T-Cell Immune Deficiency and Review of the Literature

**DOI:** 10.1007/s10875-024-01654-4

**Published:** 2024-01-16

**Authors:** Anna-Lisa Lanz, Serife Erdem, Alper Ozcan, Gulay Ceylaner, Murat Cansever, Serdar Ceylaner, Raffaele Conca, Thomas Magg, Oreste Acuto, Sylvain Latour, Christoph Klein, Turkan Patiroglu, Ekrem Unal, Ahmet Eken, Fabian Hauck

**Affiliations:** 1 Division of Pediatric Immunology and Rheumatology, Department of Pediatrics, Dr. Von Hauner Children’s Hospital, University Hospital, Ludwig-Maximilians-Universität München, Munich, Germany; 2 Faculty of Medicine, Department of Medical Biology, Erciyes University, 38030 Kayseri, Turkey; 3 Molecular Biology and Genetics Department, Gevher Nesibe Genome and Stem Cell Institute, Betul-Ziya Eren Genome and Stem Cell Center (GENKOK), Erciyes University, Kayseri, Turkey; 4 Intergen, Ankara, Turkey; 5 Faculty of Medicine, Department of Pediatrics, Division of Pediatric Hematology & Oncology, Erciyes University, Kayseri, Turkey; 6 T Cell Signalling Laboratory, Sir William Dunn School of Pathology, Oxford University, Oxford, OX2 3RE UK; 7 Laboratory of Lymphocyte Activation and Susceptibility to EBV Infection, INSERM UMR1163 Paris, France; 8 Faculty of Health Sciences, Hasan Kalyoncu University, Medical Point Hospital, Gaziantep, Turkey; 9 Munich Centre for Rare Diseases (M-ZSELMU), University Hospital, Ludwig-Maximilians-Universität München, Munich, Germany

**Correction to: Journal of Clinical Immunology**

10.1007/s10875-023-01602-8

In the original version of the article in Fig. 2C, instead of the immunoblot images for LCK and GAPDH, two orange bars were visible and the current address of Ekrem Unal has been missing in the authors information.

The original version has been corrected.

