# Supplementary figures and images for: A Novel Biallelic LCK Variant Resulting in Profound T-Cell Immune Deficiency and Review of the Literature
Source: J Clin Immunol. 2023 Dec 15;44(1):1. doi: 10.1007/s10875-023-01602-8 (PMC10724324; doi:10.1007/s10875-023-01602-8)

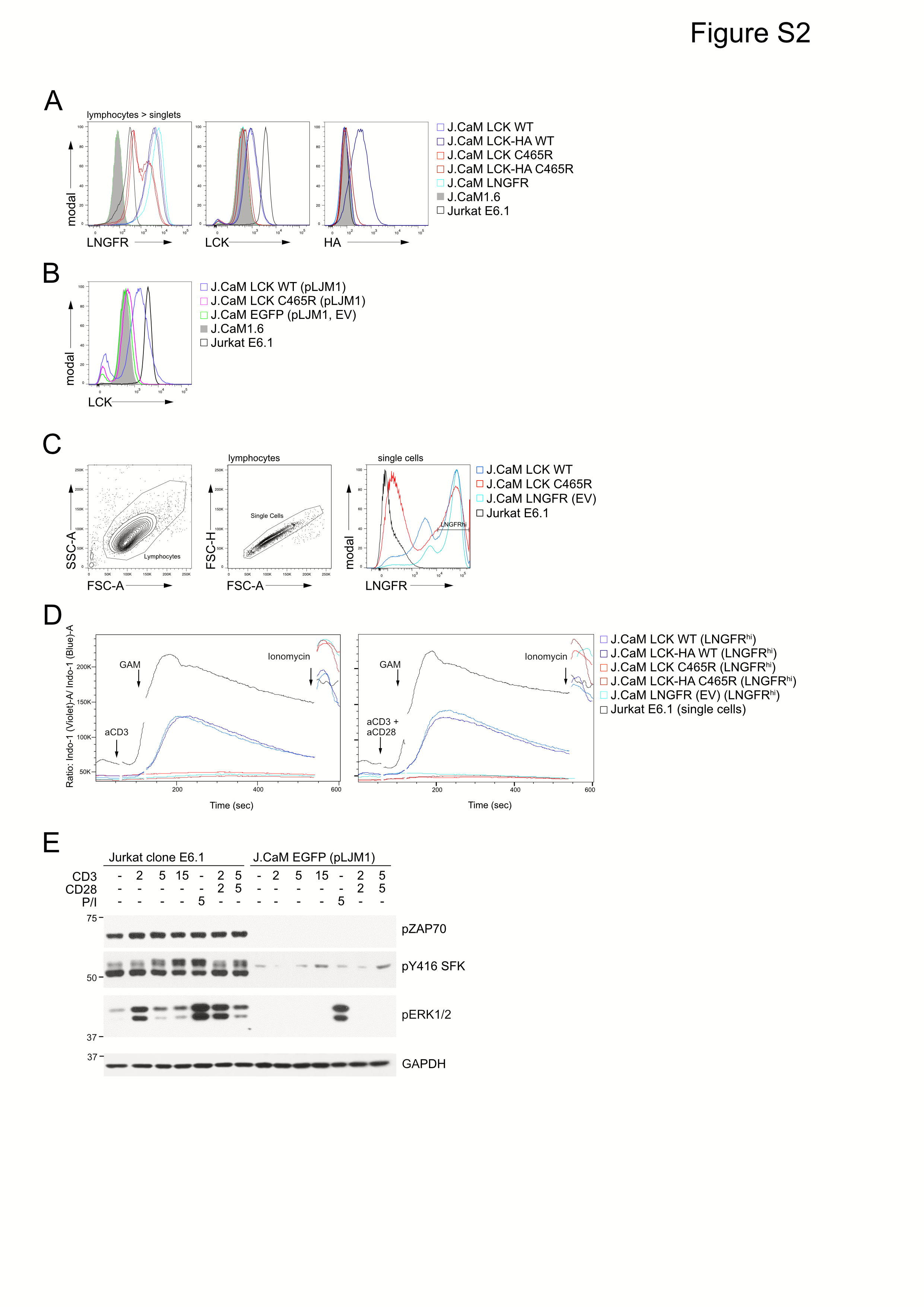

Supplement: Supplementary file 2 — Supplementary Figure S2 A Histogram overlay of flow cytometry staining of Jurkat E6.1, J.CaM1.6 and stable transduced celllines with pCDH expressing either LCK WT or LCK C465R with or without an C-terminal HA-tag or transduced with pCDH empty vector containing only the expression cassette for the extracellular domain (ED) of the low-affinity nerve growth factor receptor (LNGFR) stained with anti-LNGFR-PE, anti-LCK + secondary anti-mouse AF647 and anti-HA-AF488. B Histogram overlay of flow cytometry staining of Jurkat E6.1, J.CaM1.6 and stable transduced celllines with pLJM1 expressing either LCK WT or LCK C465R transduced with empty vector pLJM1-EGFP anti-LCK + secondary anti-mouse AF647 and anti-HA-AF488. C Gating strategy used for Ca2+-flux measurements. J.CaM1.6 cells transduced with the lentiviral plasmid that co-expresses the extracellular domain (ED) of LNGFR were gated on LNGFRhi for Ca2+-flux measurements, while for untransduced Jurkat E6.1 that do not express LNGFR-ED single cells were acquired. D Ca2+-flux measurements in J.CaM1.6 expressing LCK WT (light blue), LCK-HA (dark blue), LCK C465R (light red), LCK-HA C465R (dark red), Jurkat E6.1 (black) and J.CaM1.6 transduced with empty vector (turquoise) stimulated with anti-CD3 (left) or anti-CD3/ anti-CD28 (right). E pZAP70, pSFK (pY416), pERK1/2 immunoblots in Jurkat E6.1 cells or J.CaM1.6 transduced with empty vector pLJM1-EGFP and stimulated with either anti-CD3 (2, 5 or 15 min), anti-CD3/CD28 (2 or 5 min), PMA/ionomycin (5 min) or left untreated. (PNG 704 kb) [file 10875_2023_1602_Fig5_ESM.png]
